# Chorioretinal biomarkers in hypothalamic amenorrhea

**DOI:** 10.1007/s00417-023-06346-0

**Published:** 2024-02-26

**Authors:** Maria Cristina Savastano, Claudia Fossataro, Matteo Mario Carlà, Valentina Cestrone, Ilaria Biagini, Leonardo Sammarco, Federico Giannuzzi, Romina Fasciani, Rosanna Apa, Antonio Lanzone, Alice Diterlizzi, Martina Policriti, Enrico Di Stasio, Raphael Killian, Clara Rizzo, Stanislao Rizzo

**Affiliations:** 1 Ophthalmology Unit, “Fondazione Policlinico Universitario A. Gemelli, IRCCS,”, Largo A. Gemelli, 8, 00168 Rome, Italy; 2 Catholic University “Sacro Cuore,”, Rome, Italy; 3 Department NEUROFARBA, University of Florence, Florence, Italy; 4 Obstetric Pathology Unit, “Fondazione Policlinico Universitario A. Gemelli, IRCCS,”, 00168 Rome, Italy; 5 Department of Basic Biotechnological Sciences, Intensive Care and Perioperative Clinics Research, Catholic University of the Sacred Heart, Milan, Italy; 6 “Fondazione Policlinico Universitario A. Gemelli, IRCCS,”, 00168 Rome, Italy; 7 Ophthalmic Unit, Department of Neurosciences, Biomedicine and Movement Sciences, University of Verona, Verona, Italy; 8 Ophthalmology Unit, Department of Surgery, University Hospital, 56100 Pisa, Italy; 9 Neuroscience Institute, Italian National Research Council, CNR, Pisa, Italy

**Keywords:** Hypothalamic amenorrhea, Optical coherence tomography angiography, Choriocapillaris vascular density

## Abstract

**Purpose:**

The aim of our study was to evaluate changes in the retinal and choriocapillaris circulations in patients with hypothalamic amenorrhea.

**Methods:**

Prospective, cross-sectional observational study on 25 patients (50 eyes) diagnosed with hypothalamic amenorrhea and 25 age-matched healthy women. Optical coherence tomography angiography (OCTA) was used to evaluate the vessel density (VD) of superficial capillary plexus (SCP), deep capillary plexus (DCP), and choriocapillaris VD layers in whole 6.4 × 6.4-mm image and in fovea grid-based image. In patients’ group, systemic parameters were collected: body mass index (BMI), endometrial rhyme thickness, follicle stimulating hormone (FSH), luteinizing hormone (LH), prolactin, insulin, and cortisol.

**Results:**

SCP and DCP did not show any statistical difference when comparing patients and controls (all *p* > 0.05). Differently, choriocapillaris VD in the *whole* region showed a non-significant tendency toward higher values in the patients group in both eyes (*p* = 0.038 for right eye [RE], *p* = 0.044 for left eye [LE]). Foveal choriocapillaris VD was higher in hypothalamic amenorrhea women vs. healthy controls (66.0 ± 2.4 vs. 63.7 ± 6.6%, *p* = 0.136 for RE; 65.0 ± 2.4 vs. 61.6 ± 7.0%, *p* = 0.005 for LE). Focusing on correlation with systemic parameters, SCP and DCP *foveal* density had a medium/high effect size with endometrial rhyme, along with DCP in the *fovea* area vs. cortisol and SCP in the *whole* area vs. FSH.

**Conclusion:**

When comparing hypothalamic amenorrhea patients to healthy subjects, OCTA detected changes in the choriocapillaris layer, showing increased VD in the early stage of the systemic pathology, suggesting that microvascular “compaction” could be a first phase of hypoestrogenism adaptation.


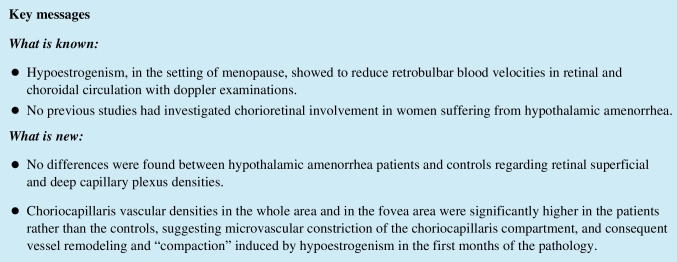


## Introduction

Amenorrhea is defined as absence or abnormal cessation of the menses, and it can be differentiated as primary or secondary entity. Primary amenorrhea consists in the absence of menses since birth. For this reason, patients do not reach menarche by the age of 15 or up to 3 years after thelarche. Causes can be represented by chromosomal irregularities (e.g., Turner syndrome) or anatomic abnormalities (e.g., Müllerian agenesis) [1]. On the other hand, secondary amenorrhea is defined as the interruption of regular menses for 3 months or the cessation of irregular menses for 6 months, excluding pregnancy. The most frequent causes of secondary amenorrhea are polycystic ovary syndrome, hypothalamic-pituitary-ovarian axis dysfunction, hyperprolactinemia, or primary ovarian insufficiency [2]. An exhaustive anamnesis in secondary amenorrhea is crucial, comprehending eating and exercise habits, presence of psychosocial stress, body weight changes, medication use, and chronic illness [3].

In most cases, amenorrhea is accompanied by severe hypoestrogenism. Low levels of estrogen manifest in multiple ways, such as low bone mineral density, vaginal and breast atrophy, infertility, and dyspareunia [4]. Moreover, in cases of hypoestrogenism, the beneficial effects on the circulatory system of these hormones should be considered. Particularly, estrogens show vasodilatory properties, modifying the production of endothelial derived substances and leading to vasodilatation through endothelial-dependent events, such as increasing nitric oxide (NO) and prostacyclin (PGI2) production and reducing endothelin (ET-1) plasma levels [5, 6]. Estrogen-mediated vasodilatation is as well caused by endothelial independent events, which seem to be calcium mediated [7]. These responses lead to a reduction in vascular resistance, which has been observed in various vascular beds such as carotid, cerebral arteries [8], and central retinal artery [9]. Several studies have investigated the features of the ocular circulation, comparing women and men, as well as fertile women and those in menopause, particularly with Doppler and pulsatility exams [10–15]. Ustymowicz et al. assessed the differences in retrobulbar blood velocities in women and men and found lower values for the short posterior ciliary artery in men compared to women [10]. Kavroulaki and colleagues, investigating choroidal blood flow in female patients of different ages, found significantly higher values in women younger than 40 years compared to women older than 55 years [11]. Further research analyzed the pulsatile ocular blood flow and pulse amplitude, which resulted significantly higher in pre-menopausal women compared to age-matched males and postmenopausal women not under hormone therapy (HT) [12]. Moreover, it has been reported that pulsatile ocular blood flow in women decreases after menopause, and suspension of estrogen replacement therapy in postmenopausal women causes reduction in ocular blood flow [13], while pre-clinical studies showed that HT increases retinal perfusion and protects retinal nerve fiber layer [16]. Consistent with these results, Toker et al. highlighted a direct relationship between serum estradiol and peak systolic velocity (PSV) and end-diastolic velocity (EDV) in the ophthalmic and central retinal arteries, measured by color Doppler imaging, suggesting a beneficial effect of estrogens on ocular hemodynamics [17]. Moreover, several works showed an increased resistivity index in both ophthalmic and short posterior ciliary arteries in postmenopausal women [18, 19]. On the other side, Wong et al. discovered that estrogen replacement therapy (ERT) seemed to exert vasoconstrictive effects in smaller retinal blood vessels, in contrast to the vasodilating effects of estrogen in bigger arteries elsewhere [20].

However, in our acknowledgment, no previous studies had investigated chorioretinal involvement in women suffering from hypothalamic amenorrhea. The recent introduction of optical coherence tomography angiography (OCTA) has allowed to deeply investigate these circulations and to obtain a post-processing quantitative analysis of vascular parameters [21]. Few researchers have used the OCTA to analyze eventual vascular changes in gynecological patients [22, 23]. Thus, the aim of our study was to evaluate eventual changes in the retinal and choriocapillaris circulations, evaluated with the OCTA, in female patients with previous diagnosis of hypothalamic amenorrhea in comparison with age- and sex-matched healthy controls. Moreover, as secondary outcome, we evaluated the correlation of systemic parameters and hormone profile obtained by blood test, with ocular biomarkers.

## Materials and methods

We conducted a prospective, cross-sectional observational study at Fondazione Policlinico Universitario A. Gemelli, IRCCS, from September 2021 to December 2022, including the Ophthalmology and Gynecology and Obstetrician Units. Women, among 18 and 34 years old, who suffered from hypothalamic amenorrhea were enrolled in the study. Amenorrhea should last at least for 6 months, to be included in the study. Age-matched healthy women were included as controls: those patients had reported regular menses in the last 6 months without the use of hormone replacement therapy (HRT). Previous full explanation, a signed informed consent was obtained from each enrolled woman. The study adhered to the Declaration of Helsinki and was approved by Ethical Committee “Università Cattolica Sacro Cuore” in Rome, Italy, with number ID: 3961.

All subjects underwent a complete ophthalmological examination, comprehensive of best corrected visual acuity (BCVA), slit-lamp examination, dilated fundus evaluation, color fundus retinography, structural optical coherence tomography (OCT), and OCTA (Solix full-range OCT, Optovue, Inc., Freemont, CA, USA). Ophthalmological exclusion criteria were high myopia or hypermetropia (± 3 diopters), diabetic retinopathy, uveitis, previous ocular surgeries, low-quality index of structural OCT, and OCTA scans (lower than 7/10), non-adherence to the study or to sign the informed consent.

Diagnosis of hypothalamic amenorrhea was derived from absence of menses for at least 6 months, a negative medroxyprogesterone acetate (MAP) test, estrogen values lower than 50 pg/mL, and FSH and LH values lower than 10 mIU/mL. Exclusion criteria were primary amenorrhea, pregnancy, usage of HRT, menopause, and other possible causes of secondary amenorrhea, such as hypophyseal ones, which were excluded performing GnRH test and prolactin dosage. For each affected patient, FSH, LH, prolactin (PRL), insulin, and cortisol values were collected, as well as the endometrial rhyme and the body mass index (BMI). None of the patients had a history of weight loss prior to the study, nor did engage in regular exercise, or suffered from anorexia nervosa, clinical depression, endocrine, or thyroid disease, nor have any other chronic medical disease. None of the patients had taken any hormonal medication for 6 months prior to the study.

### Examinations

OCT and OCTA scans were performed using the Solix full-range OCT (Optovue, Inc., Freemont, CA, USA), a ultrahigh-speed spectral domain device (version 2019 V1.0.0.317) that runs at 120.000 A-scans per second, and uses the split-spectrum amplitude-decorrelation angiography (SSADA) algorithm. Performing multiple B-scans in sequence, the software detects changes between each single scan which permits to identify the vasculature.

**Fig. 1:**
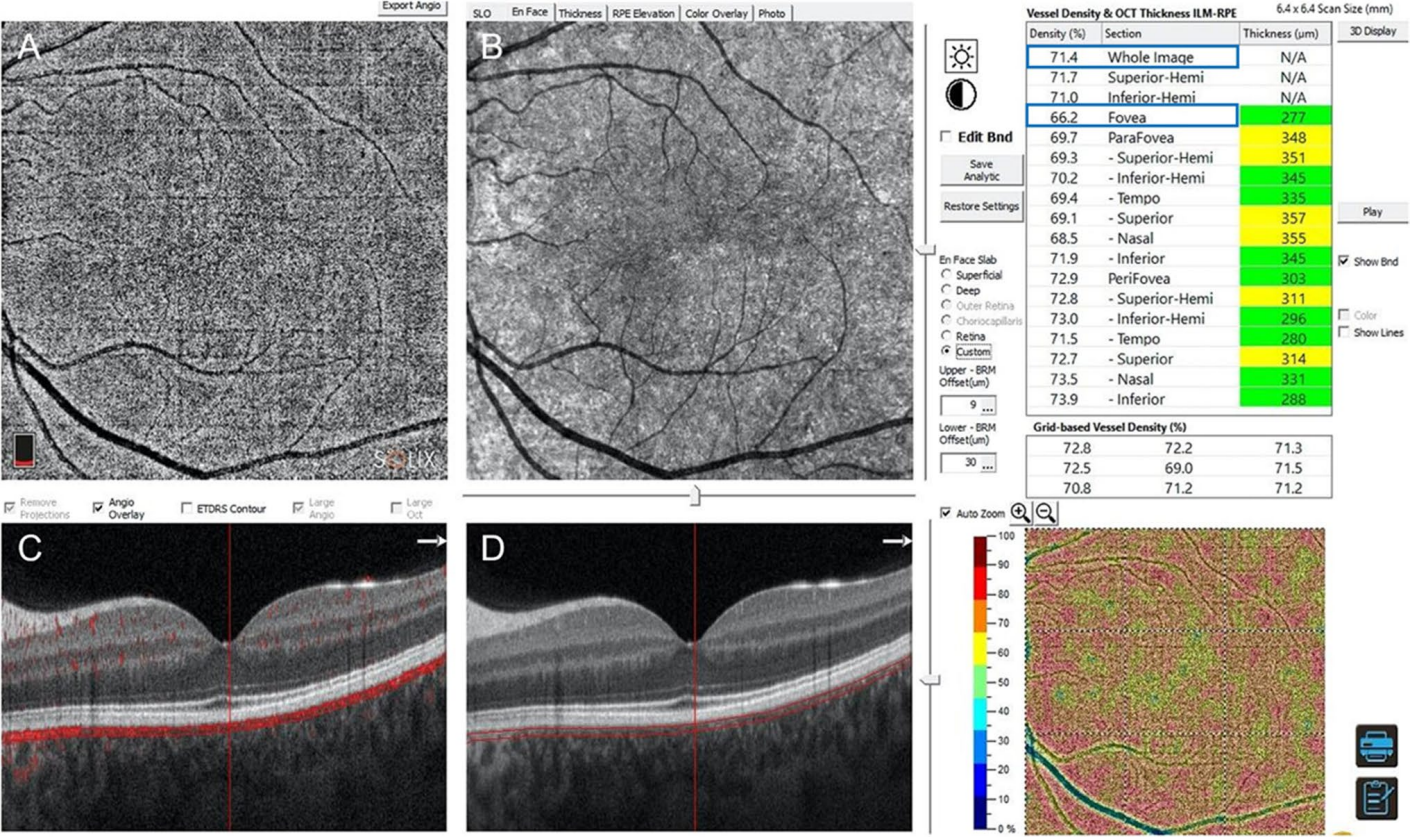
Sample of a Solix full-range OCTA analysis of the CVD using the in-built software algorithm, showing en face OCTA image (**A**) and structural OCT image (**B**) of the choriocapillaris, after setting a customized layer from 9 to 30 μm below the Bruch’s membrane, identified by the red lines in fovea-centered B-scans (**C** and **D**). The CVD of the *whole* macular area (highlighted by the upper blue rectangle) and of the *fovea* only area (in the fovea grid-based image, highlighted by the lower blue rectangle), expressed in percentage, was collected. OCTA, optical coherence tomography angiography; OCT, optical coherence tomography; CVD, choriocapillaris vascular density

Structural OCT scans were acquired to detect the single line passing horizontally and vertically in the fovea region. Successively, 6.4 × 6.4-mm OCTA scans centered on the fovea were performed. OCTA information was acquired from the AngioVue Retina software, which automatically analyses the vessel density (VD) of superficial capillary plexus (SCP), deep capillary plexus (DCP), and choriocapillaris (CVD) in *whole image* (the entire 6.4 × 6.4 mm region) and in *fovea grid-based image* (a central area automatically identified by the software)*.* Choriocapillaris vessel density (CVD) was evaluated for all patients and control group using a customized layer set from 9 μm above to 30 μm below the Bruch’s membrane and calculated using the in-built software algorithm, as showed in Fig. 1. Before processing the OCTA scans, two operators independently evaluated whether the Bruch’s membrane was correctly identified by the software. If the segmentation was not precise, the operator would have marked the layer manually (Fig. 1) [24].

### Statistical analysis

The sample size of each considered group was evaluated using the G-Power software package (version 3.1.9.7). Assuming a minimum difference of 15%, a residual standard deviation of 10%, a power of 0.08, and an alpha of 0.05 to highlight the differences, the required smallest population size was 25 patients for each group.

Statistical analysis was performed by using the Statistical Package for Social Science (SPSS), release 23.0 (IBM®, Armonk, NY). In order to assess significant differences in OCTA parameters between the patient group and healthy group, we decided to split the data of the single eye in each participant; thus, we compared all the right eyes (RE) of patients vs. controls groups and likewise for all left eyes (LE). Continuous variables were expressed as mean ± SD; categorical variables displayed as frequencies and the non-parametric Mann-Whitney *U*-test and *χ*^2^ test were used to assess significance of the differences between subgroups. Correlations were calculated with the Spearman correlation coefficient (*ρ*). After adjustment for multiple measures, a *p*-value < 0.01 was considered statistically significant. Moreover, in addiction to statistical significance, the effect size (ES) was calculated for each comparison and correlation (*d* or *r* metric, respectively, and converted in four classes—very small, small, medium, large—as appropriate) to measure the practical strength and clinical relevance of the relationship. While statistical significance, strongly influenced by the sample size, shows that an effect exists in a study, practical significance, expressed by the effect sizes and independently of the sample size, shows that the effect is large enough to be meaningful in the real world.

## Results

Twenty-five hypothalamic amenorrhea (50 eyes) and 25 age-matched healthy women (50 eyes) were included in the study. The mean age was 24.6 ± 6.0 years. Demographic characteristics and systemic parameters of the patients affected by hypothalamic amenorrhea, as well as ocular characteristics of both patients and controls, are available in Table 1.

**Table 1:** Demographic characteristics and systemic parameters of the patients affected by hypothalamic amenorrhea. Ocular parameters of both study populations

	Patients (*n* = 25)	Controls (*n* = 25)
Age (yrs)	24.7 ± 6.2	24.5 ± 5.8
Systemic parameters		
BMI	19.1 ± 1.7	
Endometrial rhyme (mm)	2.1 ± 1.7	
Amenorrhea duration (months)	17.4 ± 14.4	
Cortisol (nmol/L)	134 ± 48	
Insulin (μU/mL)	4.1 ± 2.8	
FSH0 (IU/L)	5.1 ± 2.3	
LH0 (IU/L)	2.2 ± 2.1	
PRL (mIU/L)	5.0 ± 2.6	
Ocular parameters		
BCVA (Snellen equivalent)	1.0 ± 0.0	1.0 ± 0.0
IOP (mmHg)	14.2 ± 3.6	14.6 ± 4.0
Axial length (mm)	24.2 ± 2.1	23.8 ± 1.8
Refractive error (D)	0.58 ± 0.36	0.51 ± 0.42

Data are expressed in mean ± SD

*BMI* body mass index, *FSH* follicle stimulating hormone, *LH* luteinizing hormone, *PRL* prolactin, *BCVA* best corrected visual acuity, *IOP* intraocular pressure

The VDs in either the SCP or the DCP did not show any statistical difference when comparing eyes in the controls group vs. eyes in the patients groups (all *p* > 0.05), as summarized in Table 2. In the choriocapillaris, the VD in the *whole* region showed a tendency toward higher values in the patients group in either the RE (68.7 ± 2.6% vs. 70.0 ± 1.3% in controls and patients, respectively, *p* = 0.038, *d* = 0.63, medium) or the LE (67.2 ± 3.1% vs. 68.8 ± 1.4% in controls and patients, respectively, *p* = 0.044, *d* = 0.66, medium). Moreover, in the *fovea* area of the choriocapillaris, hypothalamic amenorrhea women had a significantly higher VD (65.0 ± 2.4%) when compared to the healthy controls (61.6 ± 7.0%, *p* = 0.005, *d* = 0.64, medium) in their LE. On the other side, VD in the *fovea* area in RE showed a non-significant tendency toward the increase in patients group (63.7 ± 6.6% vs. 66.0 ± 2.4%, *p* = 0.136, *d* = 0.46, small).

**Table 2:** OCTA parameters in control and patient groups

		Controls (*n* = 25)	Patients (*n* = 25)	*p*^*Ϯ*^
SCP whole (%)	RE	51.6 ± 2.9	51.9 ± 2.0	0.772
	LE	50.8 ± 2.8	50.5 ± 2.0	0.322
SCP fovea (%)	RE	30.6 ± 5.2	30.3 ± 5.1	0.952
	LE	30.3 ± 6.0	27.9 ± 5.1	0.123
DCP whole (%)	RE	54.8 ± 5.0	57.0 ± 1.9	0.136
	LE	53.8 ± 7.6	55.6 ± 2.4	0.674
DCP fovea (%)	RE	33.7 ± 5.9	32.9 ± 6.3	0.772
	LE	34.5 ± 6.7	31.7 ± 6.6	0.136
CVD whole (%)	RE	68.7 ± 2.6	70.0 ± 1.3	0.038
	LE	67.2 ± 3.1	68.8 ± 1.4	0.044
CVD fovea (%)	RE	63.7 ± 6.6	66.0 ± 2.4	0.136
	LE	61.6 ± 7.0	65.0 ± 2.4	**0.005**

^*Ϯ*^*Mann-Whitney U-test*

*OCTA* optical coherence tomography angiography, *SCP* superficial capillary plexus, *DCP* deep capillary plexus, *CVD* choriocapillaris vessel density

Bold values are the titles of the columns

**Fig. 2:**
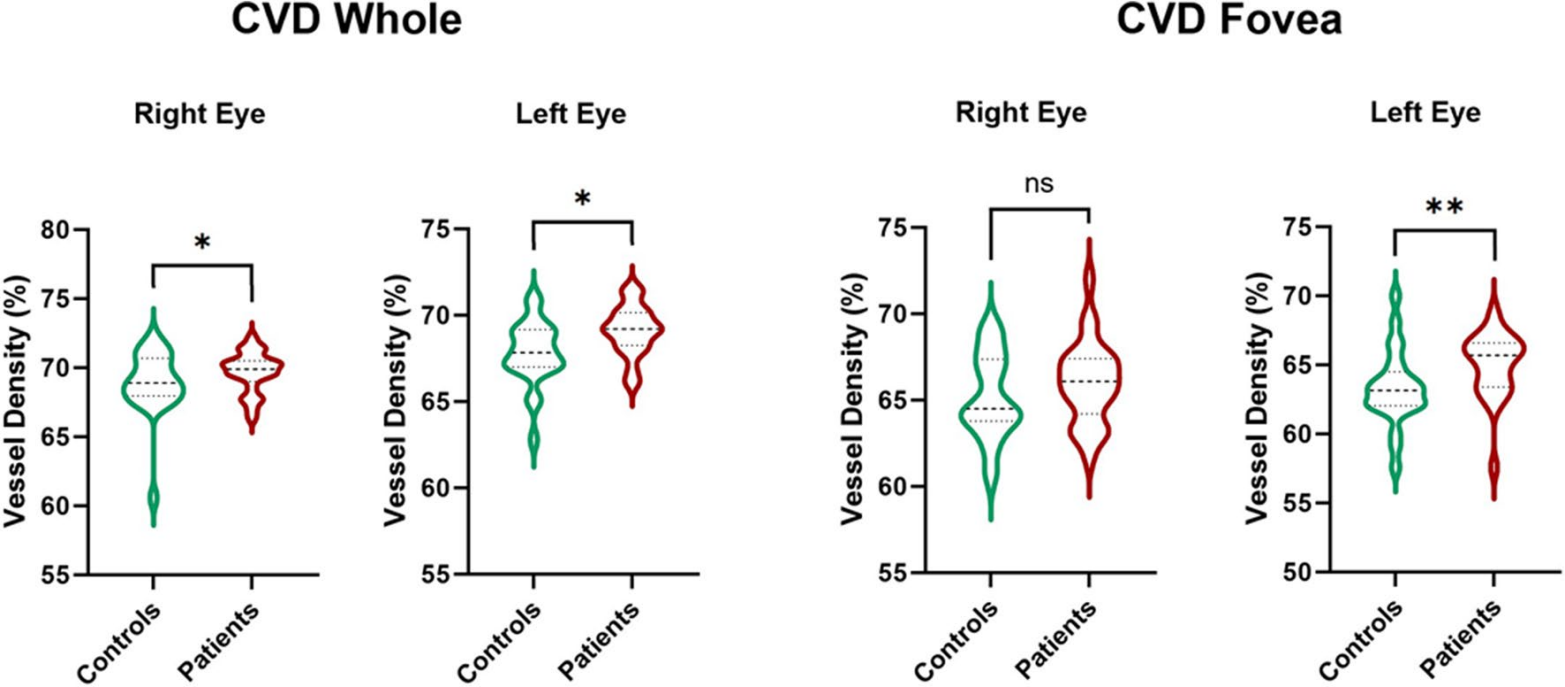
Violin plots showing differences in CVD *whole* and *fovea* between controls and patients, divided into right and left eyes. CVD, choriocapillaris vascular density; ns = no significative difference; * indicates *p* < 0.05; ** indicates *p* < 0.01

Graphical representation of CVD differences between controls and patients is available in Fig. 2.

### Correlation with systemic parameters

Correlation matrix between systemic parameters and OCTA biomarkers in hypothalamic amenorrhea women is presented in Table 3. Inverse correlations between SCP and DCP in the *fovea* area with the thickness of endometrial rhyme were found (*ρ* = −0.59 and *ρ* = −0.48 for SCP and DCP *fovea*, respectively), as well as an inverse correlation between the CVD of the *fovea* with insulin value (*ρ* = −0.67 for LE) and a direct correlation between VD of the SCP in the *whole* area and FSH_0_ values (*ρ* = 0.45 and *ρ* = 0.69 for RE and LE, respectively). In order to clinically interpret the relevance of these results, to exclude casual association between systemic biomarkers and single eye alterations and selecting only real-world implications, effect size for each correlation was calculated and converted in four classes according to *r* metrics (i.e., < 0.10 = very small, 0.10–0.29 = small, 0.30–0.49 = medium, and ≥ 0.50 = high). Only OCTA parameters showing a medium or high effect size for both eyes were deemed relevant for our clinical hypothesis. Particularly, a medium/high effect size was shown for the following correlations: (1) VDs of both the SCP and the DCP of the *foveal* area and the endometrial rhyme, (2) VD of the foveal DCP and cortisol levels, and (3) VD of the SCP and FSH_0_ values.

**Table 3:** Correlation matrix (Spearman’s correlation coefficient and significance) between OCTA and systemic parameters

			Age (yrs)	BMI	Endometrial rhyme (mm)	Amenorrhea duration (months)	Cortisol (nmol/L)	Insulin (μU/mL)	FSH_0_ (IU/L)	LH_0_ (IU/L)	PRL (mIU/L)
SCP whole (%)	RE	ρ	−0.18	0.42	−0.12	0.01	−0.27	0.40	0.45	0.17	−0.21
		*p-value*	*0.383*	*0.035*	*0.580*	*0.972*	*0.199*	*0.047*	*0.028*	*0.435*	*0.324*
	LE	*ρ*	−0.23	0.19	0.19	0.19	−0.28	0.09	0.69	0.44	0.00
		*p-value*	*0.261*	*0.351*	*0.386*	*0.369*	*0.178*	*0.682*	***< 0.001***	*0.032*	*0.984*
SCP fovea (%)	RE	*ρ*	−0.06	0.06	−0.59	0.08	0.30	0.03	0.07	0.01	0.14
		*p-value*	*0.784*	*0.790*	***0.002***	*0.690*	*0.150*	*0.886*	*0.753*	*0.967*	*0.510*
	LE	*ρ*	−0.17	0.03	−0.37	0.05	0.38	0.07	0.13	0.09	0.16
		*p-value*	*0.407*	*0.897*	*0.075*	*0.820*	*0.062*	*0.733*	*0.559*	*0.693*	*0.434*
DCP whole (%)	RE	*ρ*	−0.18	−0.05	0.17	0.11	−0.24	0.22	0.26	0.24	0.18
		*p-value*	*0.383*	*0.814*	*0.422*	*0.617*	*0.251*	*0.299*	*0.214*	*0.261*	*0.399*
	LE	*ρ*	−0.19	0.46	0.42	−0.31	−0.19	−0.09	0.35	0.47	0.28
		*p-value*	*0.365*	*0.019*	*0.043*	*0.126*	*0.374*	*0.655*	*0.097*	*0.021*	*0.179*
DCP fovea (%)	RE	*ρ*	−0.15	−0.12	−0.48	0.17	0.42	0.07	−0.10	−0.01	0.30
		*p-value*	*0.469*	*0.579*	*0.017*	*0.416*	*0.037*	*0.744*	*0.653*	*0.957*	*0.144*
	LE	*ρ*	−0.16	0.01	−0.37	0.05	0.45	0.06	0.00	0.03	0.26
		*p-value*	*0.440*	*0.975*	*0.076*	*0.795*	*0.023*	*0.772*	*0.987*	*0.871*	*0.219*
CVD whole (%)	RE	*ρ*	−0.20	0.25	0.15	0.13	−0.17	0.26	0.02	0.07	−0.13
		*p-value*	*0.328*	*0.227*	*0.476*	*0.524*	*0.413*	*0.209*	*0.909*	*0.748*	*0.539*
	LE	*ρ*	−0.44	0.40	0.13	−0.17	−0.06	0.34	0.25	0.34	0.14
		*p-value*	*0.027*	*0.049*	*0.538*	*0.409*	*0.776*	*0.101*	*0.237*	*0.104*	*0.491*
CVD fovea (%)	RE	*ρ*	0.25	−0.26	0.00	0.34	−0.28	−0.04	−0.09	−0.14	−0.22
		*p-value*	*0.237*	*0.212*	*0.984*	*0.097*	*0.172*	*0.848*	*0.687*	*0.502*	*0.282*
	LE	*ρ*	0.06	−0.14	0.14	−0.05	−0.07	−0.67	0.07	0.18	0.31
		*p-value*	*0.790*	*0.490*	*0.503*	*0.817*	*0.751*	***<0.001***	*0.750*	*0.409*	*0.131*

*ρ* = Spearman correlation

*OCTA* optical coherence tomography angiography, *SCP* superficial capillary plexus, *DCP* deep capillary plexus, *CVD* choriocapillaris vessel density, *BMI* body mass index, *FSH* follicle stimulating hormone, *LH* luteinizing hormone, *PRL* prolactin

Bold values are the titles of the columns. Italic emphasis is for *p*-value, to distinguish from the Spearman correlation coefficient (*p*). Bold-italic stands for statistical significance

## Discussion

In this cross-sectional study, we tried to point out the impact of hypothalamic amenorrhea on chorioretinal circulation. Hypothalamic amenorrhea is responsible of a long and stable decrease in estrogens levels, leading to various consequences on the human apparatus and systems, as on the circulatory system regulation. Estrogens, in fact, have a well-known role in nitric oxide (NO)–induced vasodilation, anti-atherogenesis, diminished postischemic inflammation, and anti-oxidant effects, in both macro- and micro-vascular compartments [25].

With the advent of new technologies in ophthalmologic clinical practice, researches focused on the use of OCT and OCTA to evaluate chorioretinal perfusion, rather than Doppler and pulsatility exams. Fathy et al. recently investigated the effect of postmenopausal hormonal change on optic nerve head and peripapillary perfusion [23]. They demonstrated that retinal nerve fiber layer (RNFL) thickness as well as the peripapillary VD was considerably lower in the postmenopausal group, while a significant increase in IOP was reported [23].

Recently, two studies have reported the response of retinal and choriocapillaris circulation depending on hormonal changes in women using OCTA. Kızıltunç et al. evaluated VD of macula using OCTA imaging in pregnant women, revealing that macular *whole* VD of the SCP and DCP was significantly higher compared to the control group (50.8% vs. 52.0%, *p* = 0.002 and 49.8% vs. 52.5%, *p* = 0.011, in the SCP and DCP, respectively), along with optic disc (50.8% vs. 51.9% in control and pregnant groups, respectively, *p* < 0.001) and radial peripapillary capillary VD [22]. On the other side, foveal VD of both SCP and DCP of the pregnancy group resulted significantly lower compared to the control group (18.6% vs. 20.7% and 37.0% vs. 35.3% in the SCP and DCP, respectively), associated with an increase of foveal avascular zone (FAZ) value (0.3 vs. 0.26, *p* = 0.020). They hypothesized that either hormonal changes, such as estrogens-induced vasodilation, or cardiovascular adaptations can determine an increase in retinal microvascular parameters, making it difficult to assess the specific role of estrogens in the plethora of pregnancy-induced systemic changes, thus limiting the comparability with our research.

More consistent with our work, Çetinkaya et al. recently evaluated retinal and choroidal microvascular circulation in the postmenopausal period using swept source OCTA, determining an increase in the FAZ area and a decrease in the choriocapillaris VD in the postmenopausal group compared to the age-matched menstrual group (51.% vs. 49.8%, *p* = 0.049) [26]. In particular, choriocapillaris VD was found to have a negative correlation with the duration of menopause, suggesting that a long-term hypoestrogenism significantly impacts on the microvascular density of this compartment [26].

To the best of our knowledge, this is the first study which analyzed the intraretinal plexus and the choriocapillaris circulation with OCTA in hypothalamic amenorrhea patients. The choriocapillaris network is one of the densest circulations in the human body, thus attracting the interest of researchers being subjected to several hormones and circulating factors. These findings were in line with our results where CVD was pointed out as a potential biomarker of hypothalamic amenorrhea disorder. Indeed, CVD in the whole area and in the fovea area was significantly higher (*p* = 0.005 and *p* = 0.01, respectively) in the patients rather than the controls. It is well demonstrated in cardiac microvasculature that estrogens, binding to the traditional estrogen receptors, induce arteriolar relaxation and flow-induced dilatation. On the other side, decreasing estrogen levels causes impairment in endothelial function due to decreased NO production, increased oxidative stress, and proinflammatory cytokines production, along with abnormal vascular remodeling and microvascular constriction [27]. Starting on this assumption, we speculate that the increased CVD we found in hypothalamic amenorrhea patients derives from microvascular constriction of the choriocapillaris compartment and consequent vessel remodeling and “compaction.” Moreover, we hypothesize that this microvascular “compaction” could be a first phase of hypoestrogenism adaptation, since the mean duration of amenorrhea in our patients was 17 months. In fact, long-term hypothalamic amenorrhea is strongly associated with premature cardiovascular risk which increases over time, thus suggesting the existence of more advanced stages of microvascular impairment even in chorioretinal circulation [4].

From a systemic point of view, the correlation of ocular microvascular parameters and gynecologic parameters showed some correlations of retinal VD with the endometrial rhyme and FSH values, but these results were difficult to interpret since several multifactorial mechanisms are implied and may act as confounders in these correlations. Surely, longitudinal studies could be helpful to point out significant associations between systemic parameters evolution and ocular microvascular changes.

Our study has some limitations, such as the small sample size, the monocentric data gathering, and the cross-sectional setting. Future collaborative research are needed to explore more in depth the role of ocular parameters, as predictive or prognostic biomarkers of systemic microvascular impairment in this condition. We indeed plan to follow our patients over time with a multidisciplinary team, in order to define the evolution of this pathology and, meantime, to evaluate the effect of possible substitutive therapy on ocular microvascular biomarkers.

In conclusion, OCTA can play a significant role in the diagnosis of microvascular alterations, which appear to be mostly localized in the choriocapillaris layer, in hypothalamic amenorrhea patients, offering new insights for longitudinal evaluation of ocular parameters.

## Data Availability

The data that support the findings of this study are available from the corresponding author, upon reasonable request.
